# Intravitreal Dexamethasone Implant (Ozurdex) for Refractory Macular Edema Secondary to Retinitis Pigmentosa

**DOI:** 10.4274/tjo.60437

**Published:** 2016-08-15

**Authors:** Nurgül Örnek, Kemal Örnek, İnci Elif Erbahçeci

**Affiliations:** 1 Kırıkkale University Faculty of Medicine, Department of Ophthalmology, Kırıkkale, Turkey

**Keywords:** Macular edema, retinitis pigmentosa, dexamethasone implant

## Abstract

Macular edema (ME) in retinitis pigmentosa (RP) often impairs central vision dramatically. A 41-year-old woman diagnosed with RP was referred to our outpatient clinic due to severe visual deterioration in both eyes. The patient was treated with topical carbonic anhydrase inhibitors, topical corticosteroids and intravitreal triamcinolone acetonide injections, but her ME recurred. Intravitreal 0.7 mg dexamethasone implant (Ozurdex, Allergan) was administered into both eyes without complications. On the fourth day after both injections, visual acuity improved and ME almost totally resolved. No recurrence was observed at follow-up six months later.

## INTRODUCTION

Macular edema (ME) in retinitis pigmentosa (RP) often impairs central vision dramatically. ME has been shown in at least one eye in 32% of patients and in both eyes in 18% of patients in a recent optical coherence tomography (OCT) study.^1^ Although the pathogenesis of RP-related ME has not been fully established, general inflammatory response to degenerating photoreceptors and retina pigment epithelium, defective blood-aqueous barrier, and autoimmune process (antiretinal antibodies) have been proposed as the possible causes.^2,3,4^

Treatment options include carbonic anhydrase inhibitors, corticosteroids, anti-vascular endothelial growth factor (anti-VEGF) agents, grid laser photocoagulation and vitrectomy.^5,6,7,8,9^ Off-label intravitreal injection of triamcinolone has also been found to be effective.^4^ A sustained-release dexamethasone implant is available for the treatment of ME secondary to retinal vein occlusion and in recent years it has been shown to have favorable results in the treatment of ME secondary to RP.^5,6^

Here, we report a case with bilateral refractory ME secondary to RP which dramatically improved within the first week following dexamethasone implant.

## CASE REPORT

A 41-year-old woman diagnosed with RP was referred to our outpatient clinic 3 years ago due to severe visual deterioration in both eyes. She had refractory ME secondary to RP for about 12 years. The patient had been unresponsive to both topical carbonic anhydrase inhibitors and topical corticosteroids.

**Figure 1:**
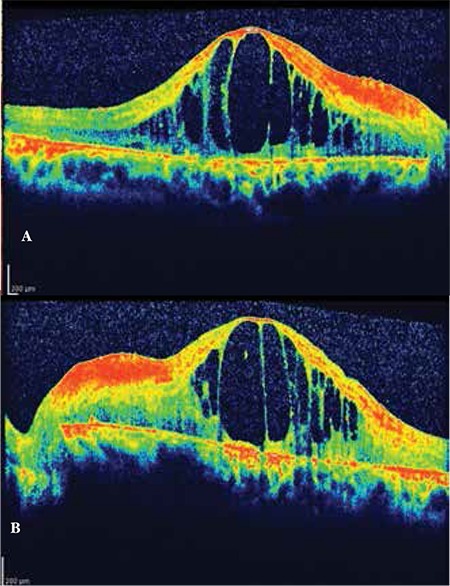
Optical coherence tomography imaging shows severe macular edema on initial admission (A: right eye, B: left eye)

**Figure 2:**
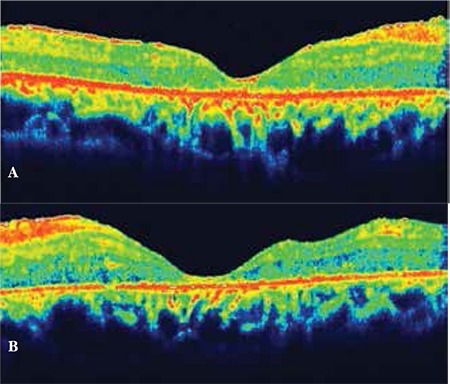
Macular edema resolved after the first triamcinolone acetonide injection (A: right eye, B: left eye)

On initial examination, anterior segment details were within normal ranges and intraocular pressure was below 21 mmHg in both eyes. Visual acuity was counting fingers (CF) in both eyes. She had severe ME with RP in both eyes (Figure 1). The diagnosis was confirmed using OCT and fundus fluorescein angiography. Full-field electroretinogram showed typical delays in both rod and cone b-wave implicit times. She received intravitreal triamcinolone acetonide (TA) injections in both eyes. Following the injections, visual acuity had increased to 20/320 in oculus dexter (OD) and 20/400 in oculus sinister (OS) according to Snellen chart (Figure 2). Eight months later, ME recurred and second TA injections were performed. Between the injections, the patient was treated with oral acetozolamide (Diazomid 250 mg, Sanofi-Aventis, Turkey) 125 mg twice daily. Over the follow-up period of 1 year, posterior subcapsular cataract developed in both eyes and vision decreased to preinjection levels. The patient underwent bilateral phacoemulsification surgery with intraocular lens implantation and intravitreal TA injections. Visual acuity improved from CF to 20/320 in OD but remained CF in OS.

**Figure 3:**
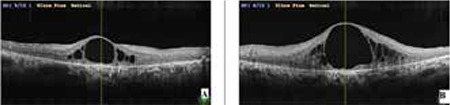
Severe macular edema prior to dexamethasone implant injection (A: right eye, B: left eye)

**Figure 4:**
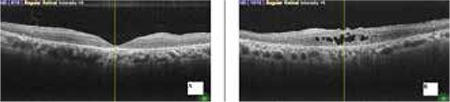
Macular edema had almost totally resolved 4 days after dexamethasone implant injection (A: right eye, B: left eye)

**Figure 5:**
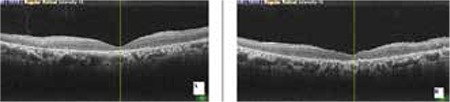
No macular edema in both eyes at third month (A: right eye, B: left eye)

**Figure 6:**
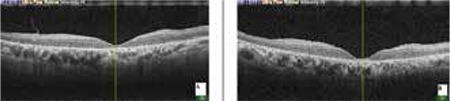
No recurrence at 6 months in both eyes (A: right eye, B: left eye)

Four months later, visual acuity decreased again to CF in OD. OCT revealed severe cystoid ME (CME) in both eyes, central foveal thickness was 613 µm in OD and 1071 µm in OS (Figure 3). With informed consent, intravitreal 0.7 mg dexamethasone implant (Ozurdex, Allergan, USA) was administered as an off-label treatment to both eyes without complications on separate days. On the fourth day after injections, visual acuity improved to 20/320 in OD and 20/800 meter in OS, and the ME had almost completely resolved (Figure 4). No recurrence was observed during the follow-up examination 6 months later (Figures 5 and 6).

## DISCUSSION

There are several recent reports of intravitreal injection of dexamethasone implant (Ozurdex) for the treatment of ME secondary to RP.^10,11,12,13^ Srour et al.^10^ administered intravitreal dexamethasone implant in 3 patients with mean central macular thickness (CMT) of 443±185 µm (range 213-619 µm) and mean visual acuity of 20/160 (20/50-20/200) at baseline. One month after dexamethasone implantation, mean CMT improved to 234±68 µm and mean BCVA improved to 20/100. Saatci et al.^11^ reported a case with bilateral ME secondary to RP. Visual acuity of the patient was 2/10 in both eyes and he had been under topical dorzolamide treatment 3 times a day for nearly a year without any change in VA. One week after the injection his visual acuity improved to 4/10 and ME resolved. Buchaim et al.^12^ also reported successfully using intravitreal dexamethasone implant for the treatment of ME due to RP. Very recently, Ahn et al.^13^ treated a 24-year-old patient with RP who developed CME in both eyes that was refractory to oral acetazolamide and intravitreal bevacizumab treatment. Despite a second intravitreal dexamethasone implant injection, CME recurred in both eyes 6 months later. The intravitreal dexamethasone implant may be useful for CME in patients with RP, but its efficacy seems to be limited over time. In our case, visual acuity improvement was almost the same with intravitreal triamcinolone acetonide injection and intravitreal dexamethasone implant. No recurrence was observed up to six months following the dexamethasone implant injection. ME and visual loss were more severe (CMT, 1071 µm) in the left eye due to damage to the external limiting membrane and photoreceptor layers. Despite severe structural changes in the macular area, ME resolved almost totally and visual acuity improved from CF to 20/800 at the 4^th^ day visit, which made the patient very satisfied.

## CONCLUSION

Although the follow-up period of our patient was short, long-lasting refractory ME secondary to RP may respond very rapidly to intravitreal dexamethasone implant with satisfactory results both for the patient and ophthalmologist. Further studies with larger sample size and longer durations are needed to clarify this issue.

### Ethics

Informed Consent: It was taken.

Peer-review: Externally peer-reviewed.
